# Identification and diagnostic potential of hsa_circ_101303 in colorectal cancer: unraveling a regulatory network

**DOI:** 10.1186/s12885-024-12458-5

**Published:** 2024-06-01

**Authors:** Ke-zhi Li, Xiao-min Liao, Si-qi Li, Hao-tang Wei, Zhi-jian Liang, Liu-xin Ge, Su-fang Zhou, Bang-li Hu

**Affiliations:** 1 Department of Research, Guangxi Medical University Cancer Hospital, No. 71 Hedi Road, Nanning, 530021 China; 2 Department of Gastrointestinal Surgery, Third Affiliated Hospital of Guangxi Medical University, Nanning, 530031 China; 3 Department of Biochemistry and Molecular Biology, School of Basic Medical Sciences, Guangxi Medical University, No. 22 Shuangyong Road, Nanning, 530021 China; 4 Key Laboratory of the Ministry of Education Project for Early Prevention and Treatment of Regional High-Risk Tumors & Key Laboratory of Biological Molecular Medicine Research, Guangxi Medical University, Nanning, 530021 China

**Keywords:** Colorectal cancer, Circular RNA, Hsa_circ_101303, Diagnostic biomarker, ceRNA network

## Abstract

**Background:**

The role of novel circular RNAs (circRNAs) in colorectal cancer (CRC) remains to be determined. This study aimed to identify a novel circRNA involved in CRC pathogenesis, assess its diagnostic value, and construct a regulatory network.

**Methods:**

Differential expression analysis was conducted using circRNA datasets to screen for differentially expressed circRNAs. The expression of selected circRNAs was validated in external datasets and clinical samples. Diagnostic value of plasma circRNA levels in CRC was assessed. A competing endogenous RNA (ceRNA) network was constructed for the circRNA using TCGA dataset.

**Results:**

Analysis of datasets revealed that hsa_circ_101303 was significantly overexpressed in CRC tissues compared to normal tissues. The upregulation of hsa_circ_101303 in CRC tissues was further confirmed through the GSE138589 dataset and clinical samples. High expression of hsa_circ_101303 was associated with advanced N stage, M stage, and tumor stage in CRC. Plasma levels of hsa_circ_101303 were markedly elevated in CRC patients and exhibited moderate diagnostic ability for CRC (AUC = 0.738). The host gene of hsa_circ_101303 was also found to be related to the TNM stage of CRC. Nine miRNAs were identified as target miRNAs for hsa_circ_101303, and 27 genes were identified as targets of these miRNAs. Subsequently, a ceRNA network for hsa_circ_101303 was constructed to illustrate the interactions between the nine miRNAs and 27 genes.

**Conclusions:**

The study identifies hsa_circ_101303 as a highly expressed circRNA in CRC, which is associated with the progression of the disease. Plasma levels of hsa_circ_101303 show promising diagnostic potential for CRC. The ceRNA network for hsa_circ_101303 provides valuable insights into the regulatory mechanisms underlying CRC.

## Background

According to a recent report from GLOBOCAN 2020 [1], colorectal cancer (CRC) is the third most frequent malignancy and the fourth most common cause of cancer-related death. Several cytokines and environmental factors have been demonstrated to contribute to the carcinogenesis of CRC. Over the past several decades, the processes underlying the etiology and development of CRC have been thoroughly explored. Circular RNAs (circRNAs) have been known for decades, but they were primarily thought of as by-products of mRNA splicing or as useless trash RNA [2]. Hansen et al. [3] recently discovered that circRNAs have the ability to attach to miRNAs in a competitive manner, inhibiting their expression while enhancing the expression of the miRNAs’ downstream target genes. Since then, a large number of researchers have investigated the role played by circRNAs in both physiological and pathological processes that underlie a number of disorders [4].

With the advancement of detection techniques, such as circRNA microarray screening and high-throughput RNA sequencing, the association between thousands of circRNAs and disease pathogenesis has been established. This suggests the potential of circRNAs to serve as crucial biomarkers for disease pathogenesis, progression, therapeutic response, and prognosis [5]. circRNAs have also been shown to be associated with the development of several cancers, including CRC [6]. Recent studies have uncovered several circRNAs involved in the regulation of CRC development and progression. For instance, Yan et al. demonstrated that circ_0065378 upregulates the expression of tumor suppressor candidate 1 by competitively binding with miR-4701-5p, thus alleviating CRC progression [7]. Upregulation of CCND2 through the dual IGF2BP3/miR-375 pathway has been linked to cell growth promotion in CRC by hsa_circ_0000231 [8]. In addition, hsa_circ_0007507 [9], hsa_circ_0001821 [10] have identified as novel diagnostic and prognostic biomarkers for CRC.

**Fig. 1:**
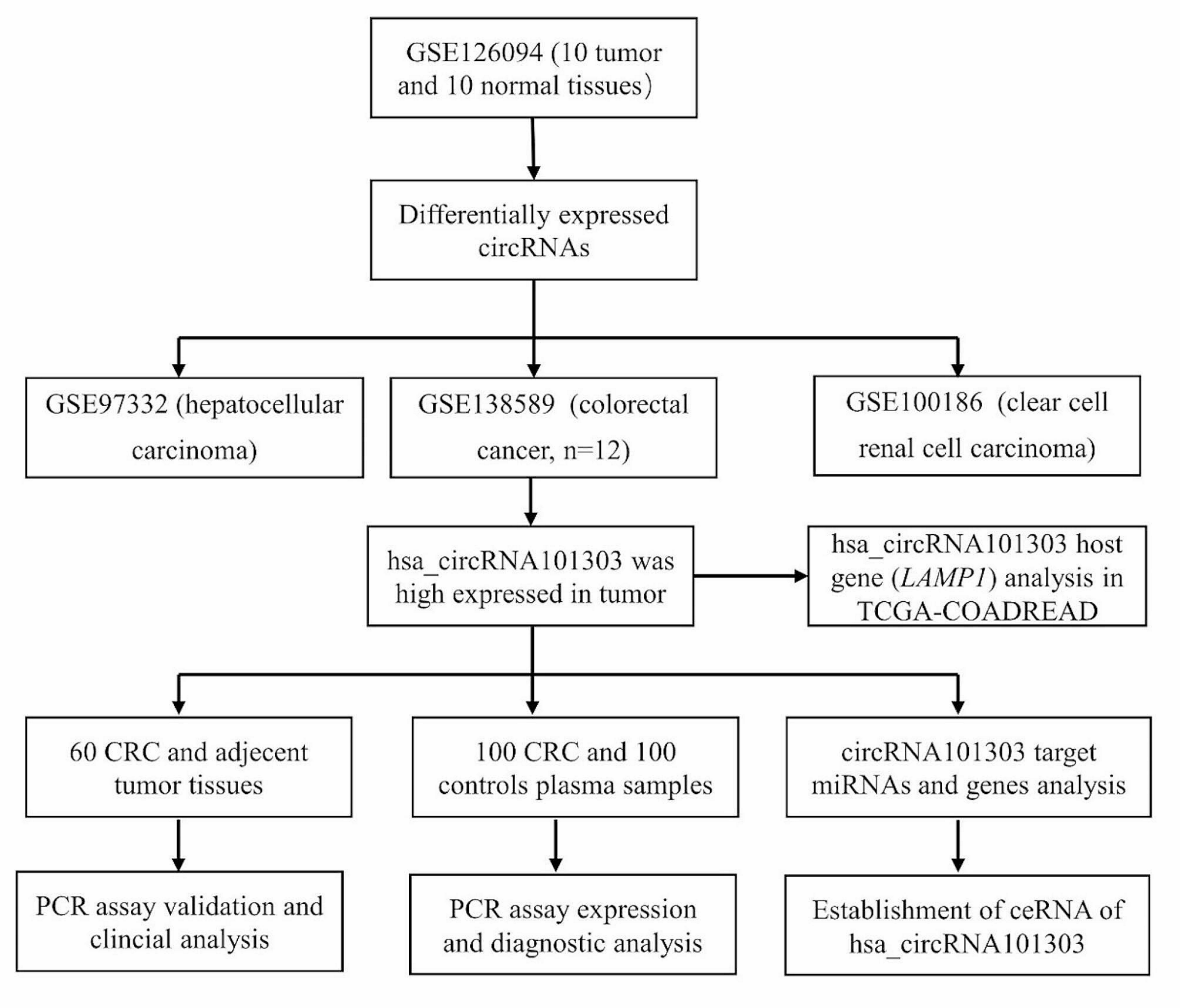
The flow diagram of present study

Although several circRNAs have been found to be associated with CRC, many others still need to be investigated. In this study, we analyzed a circRNA microarray dataset and screened circRNAs associated with the development of CRC. Then, we selected a significant differentially expressed circRNA (hsa_circ_101303) and validated its expression using an external cohort and clinical samples. Next, we explored its plasma levels in diagnostic value on CRC. We also explored the clinical significance of this circRNA and its host gene in CRC. Finally, we constructed a competing endogenous RNA (ceRNA) network using a TCGA dataset for this circRNA, to further investigate its potential role in the pathogenesis of CRC. Our findings provide important information of the role of this circRNA in CRC. The flow diagram is shown in Fig. 1.

## Material and method

### The acquisition of circRNA datasets in CRC

We conducted a search in the Gene Expression Omnibus (GEO) database to identify datasets containing circRNA profiles in CRC. Using GPL19978 as the probe platform, we retrieved three datasets: GSE126094 [11] (*n* = 20), GSE138589 [12] (*n* = 12) and GSE142837 [13] (*n* = 10). We also downloaded two datasets (GSE97332 [14] and GSE100186 [15]), which employed the same probe as the GPL19978 platform. Raw data pertaining to circRNA expression were extracted from the GEO datasets and subsequently preprocessed using the RMA algorithm for background adjustment prior to analysis. Furthermore, we obtained the TCGA-COADREAD dataset (featuring count data) from the TCGA database via the GDC hub (https://gdc.xenahubs.net). For the GEO datasets, we performed robust multichip average (RMA) preprocessing with the “affy” package to adjust the background and perform quantile normalization before analysis. Differential expression analysis was then carried out on the GSE126094 dataset using the “limma” package to identify differentially expressed circRNAs (DEcircRNAs) between tumor and control tissues.

### Selection of target circRNA associate with CRC

To identify target circRNAs associated with CRC, we initially conducted a screening for DEcircRNAs in a GEO dataset comprising CRC and normal tissues. Subsequently, the identified DEcircRNAs were further scrutinized across multiple external GEO datasets specific to CRC to pinpoint the target circRNA. The expression level of the selected circRNA was then corroborated using clinical CRC tissue samples. Employing the Statulator online analytical tool (https://statulator.com/) with the “Sample Size Calculator for Comparing Two Independent Means” feature. This calculation yielded a minimum sample size of 36 to ensure statistical significance.

### The collection of CRC tissues and blood samples

From January 2015 to December 2017, a total of sixty CRC samples and their corresponding adjacent tissues were collected from a hospital. The samples were obtained following surgical resection and promptly snap frozen before being stored at -80˚C for subsequent analysis. Only samples with histological confirmation of CRC were selected, and cases with severe major organ dysfunction or prior cancer chemotherapy were excluded. In addition to the tissue samples, 115 plasma samples from CRC patients and 73 plasma samples from non-cancer patients were collected during the same period. A peripheral venous blood sample of 10 mL was collected using an EDTA anticoagulant tube. The plasma samples were stored at -80℃ until further processing. Detailed clinical characteristics of both the tissue and plasma samples are provided in Table 1.

**Table 1:** Clinical significance of hsa_circ_101303 in CRC

	Number (*N* = 60)	High-expression (*N* = 30)	low-expression (*N* = 30)	*P* value
Age	63.9 (13.5)	64.8 (12.2)	62.9 (14.8)	0.591
Gender				0.295
Female	35 (58.3%)	15 (50.0%)	20 (66.7%)	
Male	25 (41.7%)	15 (50.0%)	10 (33.3%)	
Location				0.252
CRC	43 (71.7%)	24 (80.0%)	19 (63.3%)	
Rectal cancer	17 (28.3%)	6 (20.0%)	11 (36.7%)	
Site				0.080
Right colon	23 (38.3%)	10 (33.3%)	13 (43.3%)	
Left colon	20 (33.3%)	14 (46.7%)	6 (20.0%)	
Rectal	17 (28.3%)	6 (20.0%)	11 (36.7%)	
T stage				0.161
T2	6 (10.0%)	1 (3.33%)	5 (16.7%)	
T3	26 (43.3%)	12 (40.0%)	14 (46.7%)	
T4	28 (46.7%)	17 (56.7%)	11 (36.7%)	
N stage				0.008
N0	33 (55.0%)	21 (70.0%)	12 (40.0%)	
N1	14 (23.3%)	2 (6.67%)	12 (40.0%)	
N2	13 (21.7%)	7 (23.3%)	6 (20.0%)	
M stage				0.024
M0	48 (80.0%)	20 (66.7%)	28 (93.3%)	
M1	12 (20.0%)	10 (33.3%)	2 (6.67%)	
Tumor stage:				<0.001
I	6 (10.0%)	1 (3.33%)	5 (16.7%)	
II	24 (40.0%)	17 (56.7%)	7 (23.3%)	
III	18 (30.0%)	2 (6.67%)	16 (53.3%)	
IV	12 (20.0%)	10 (33.3%)	2 (6.67%)	
Grade				0.832
Low	6 (10.0%)	4 (13.3%)	2 (6.67%)	
Middle	52 (86.7%)	25 (83.3%)	27 (90.0%)	
High	2 (3.33%)	1 (3.33%)	1 (3.33%)	
Ki_67	65.8 (13.1)	63.5 (13.5)	68.0 (12.6)	0.186
P53	1.18 (1.02)	1.17 (1.05)	1.20 (1.00)	0.900
CEA	1.83 (1.08)	1.67 (1.09)	2.00 (1.05)	0.233

### RT-qPCR assay for the expression of circRNA

Total RNA was isolated from CRC tissues using the NucleoZOL isolation kit (Macherey-Nagel, Düren, Germany), following the manufacturer’s protocol. For circRNA analysis, complementary DNA (cDNA) was synthesized using the Prime ScriptTM RT Master Mix (Perfect Real Time) and reagent kit (TaKaRa Bio, Shiga, Japan). The expression levels of circRNAs were determined by RT-qPCR using the ChamQ Universal SYBR qPCR master mix (Vazyme, Nanjing, China), with human GAPDH serving as the internal control. Primers for the ampl ification of circRNAs were synthesized by GENESEED Biotechnology (Guangzhou). The human *GAPDH* primers were as follows: (forward) 5′- AGA AGG CTG GGG CTC ATT TG-3′ and (reverse) 5′- GCA GGA GGC ATT GCT GAT GAT-3′. The hsa_circ_101303 primers were as follows: (forward) 5′-CCT GAT GTT TTG CCG TGG CT-3′ and (reverse) 5′-CGG CTC GGC TCC CCG CAA AA-3′. The levels of circRNA expression were then quantified by the 2-ΔΔCt method.

### Prediction of circRNA target miRNAs and the target genes for miRNAs

The identification of miRNAs targeted by the selected circRNAs was screened by the CircInteractome online tool (https://circinteractome.irp.nia.nih.gov/) [16]. This tool provides a platform for mapping miRNA binding sites on reported circRNAs, thereby offering reliable predictions concerning circRNA-miRNA interactions. To extend our investigation into the genetic implications of these interactions, we sought to identify the gene targets of the predicted miRNAs. This was accomplished using the miRWalk online tool [17] (version 2, http://mirwalk.umm.uni-heidelberg.de/). miRWalk is distinguished by its comprehensive database that includes potential binding site interactions across the entirety of gene sequences and miRNAs, based on the TarPmiR algorithm. This approach combines multiple predictive models, including Targetscan, miRDB, and miRTarBase, the latter providing a repository of experimentally validated miRNA-target interactions. These methodologies together enhance the accuracy of the predictions, offering a comprehensive analysis of potential target genes.

### Construction of the ceRNA network

We developed a competitive endogenous RNA (ceRNA) network based on the methodology outlined in our previous study [18], which encapsulates the intricate interactions among circRNAs, miRNAs, and genes. Initially, differentially expressed miRNAs (DEmiRNAs) between tumor and normal CRC tissues were identified utilizing the TCGA-COADREAD dataset, maintaining a significance threshold of *P* < 0.05. Subsequently, the identified DEmiRNAs were matched with target miRNAs predicted by the CircInteractome online tool to establish circRNA-miRNA pairs. Next, the targeted genes of miRNAs were determined using the miRWalk online tool with a confidence score exceeding 0.95. Differentially Expressed Genes (DEGs) between tumor and control CRC tissues were identified from the TCGA-COADREAD dataset with criteria of log-fold change value > 2 and a P value < 0.01. Subsequently, the targeted genes of miRNAs and the DEGs were intersected to obtain miRNA-gene pairs. By integrating these circRNA-miRNA pairs and miRNA-gene pairs, the interactions within the ceRNA network were established. For effective visualization and presentation of the ceRNA network, these circRNA-miRNA-gene interactions were integrated into Cytoscape software (version 3.8.2, San Diego, CA). This robust software enabled us to construct and visually represent the complex relationships between circRNAs, miRNAs, and genes within the ceRNA network. To visualize the ceRNA network using Cytoscape software, two essential files were required. One file contained the circRNA-miRNA-gene interaction data, structured in two columns with miRNAs in one column and circRNA along with genes in another column. The other file comprised annotation data that annotated the type of each node. These two files were imported into the software to generate the ceRNA network, utilizing a circular layout format for node arrangement.

### Statistical analysis

The statistical analysis was performed employing suitable tests based on the distribution of variables, with normality assessed using the Shapiro-Wilk normality test. For normally distributed variables, differences between two groups were evaluated using the Student’s t-test. In cases where variables deviated from normal distributions, the Mann-Whitney U test was utilized. When comparing more than two groups and assuming normal distribution, one-way analysis of variance (ANOVA) was employed; otherwise, the Kruskal–Wallis test was used. Categorical variables were analyzed using the Chi-squared test or Fisher’s Exact Test. To ascertain the diagnostic efficacy of the circRNA, Receiver Operating Characteristic (ROC) curves were constructed utilizing data from tumors and controls. The corresponding Area Under the Curve (AUC) was computed to gauge the discriminative power. AUC values below 0.6 were classified as poor, those within the range of 0.7 to 0.8 were considered moderate, and values exceeding 0.8 were deemed good. All statistical analyses were executed using the R language (version 4.1.1). A significance level of two-tailed *P* < 0.05 was used to for determining statistical significance.

## Results

### The identification of DEcircRNAs from a circRNA dataset

**Fig. 2:**
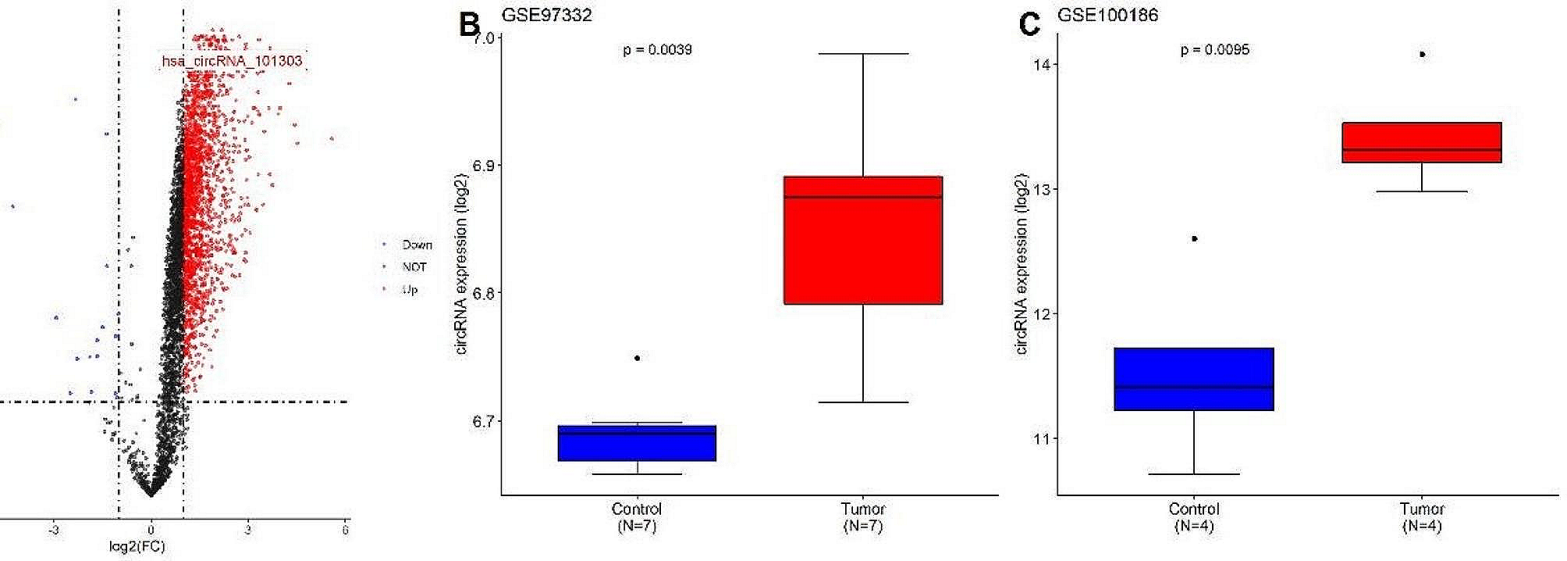
**(A)** Volcano plot of the differentially expressed circRNA in GSE126094 dataset. Each dot in the plot represents a gene, red dot represents genes that are up-regulated, blue dot corresponds to genes that are down-regulated, black dot represents genes with statistically insignificant differences in expression levels; Expression of hsa_circ_101303 in **(B)** GSE97332 dataset and **(C)** GSE100186 dataset. Data was shown as mean ± SD.

The analysis of the GSE126094 dataset led to the identification of 243 DEcircRNAs between 10 CRC tumor tissues and 10 normal tissues, applying criteria of |logFC| > 2 and *P* < 0.01. Notably, hsa_circ_101303 was found to be significantly upregulated in tumor tissues in comparison to normal tissues (Fig. 2A). Further analyses of the GSE97332 and GSE100186 datasets revealed elevated expression of hsa_circ_101303 in hepatocellular carcinoma (HCC) and clear cell renal cell carcinoma (CCRC) (*P* < 0.05, Fig. 2B-C), These results underscore the potential oncogenic role of hsa_circ_101303 in various cancer types and hinting at its involvement in tumorigenesis.

### Validation of hsa_circRNA101303 in independent datasets and clinical samples

**Fig. 3:**
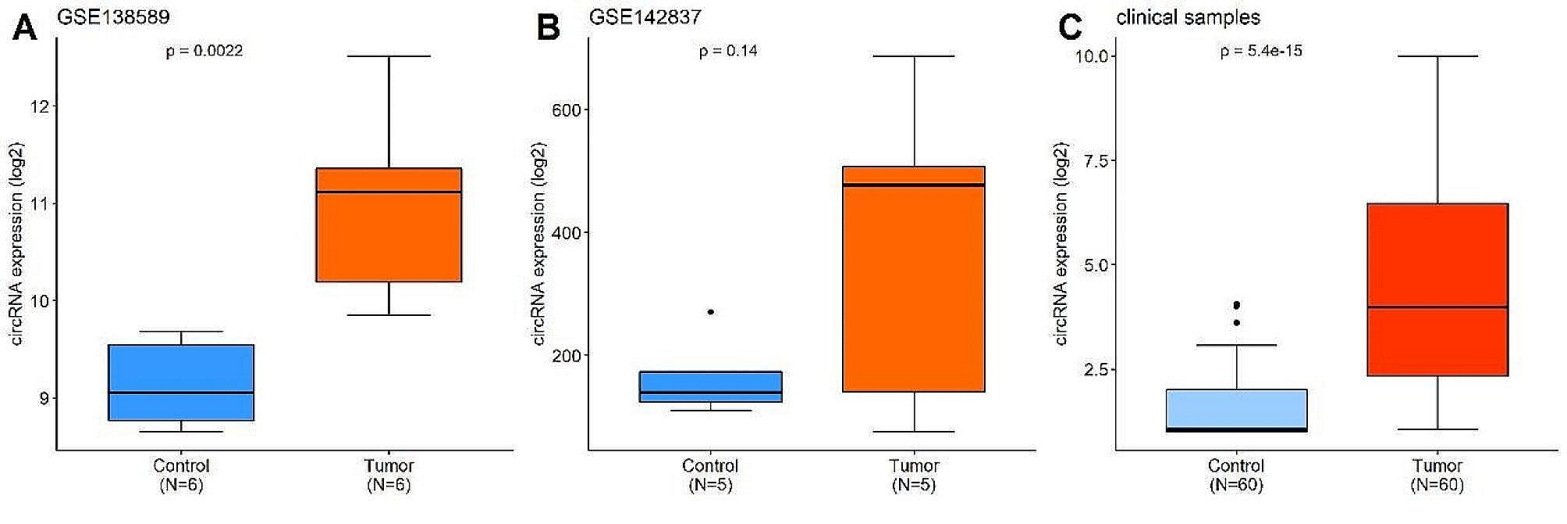
Expression of hsa_circRNA101303 in CRC tissues. Comparison of hsa_circRNA101303 expression between CRC tumor tissues and control tissues in **(A)** GSE138589 dataset; **(B)** GSE142837 dataset; **(C)** Clinical samples. Data was shown as mean ± SD.

The expression of hsa_circ_101303 was validated in two additional datasets. Analysis of the GSE138589 dataset, encompassing 6 CRC tumor tissues and 6 normal tissues, demonstrated a significant upsurge in the expression of hsa_circ_101303 in tumor tissues (*P* < 0.05). However, in the GSE142837 dataset, comprising 5 CRC tumor tissues and 5 normal tissues, the increased expression of hsa_circ_101303 in tumor tissues did not reach statistical significance (*P* > 0.05). Further validation through RT-qPCR assay on 60 CRC tumor tissues and corresponding paired normal tissues confirmed the heightened expression of hsa_circ_101303 in CRC tissues (*P* < 0.05; Fig. 3), reinforcing the evidence of its upregulation in CRC.

### Association of hsa_circ_101303 with clinical parameters in CRC

We further examined the relationship between hsa_circ_101303 expression and clinical parameters in CRC utilizing clinical sample data. As shown in Table 1, by using the median expression value as a threshold, no statistically significant correlations were discovered between hsa_circ_101303 expression and patient gender, tumor location, or T stage (*P* > 0.05). Nevertheless, significant associations were observed between its expression and the N stage, M stage, and overall tumor stage (*P* < 0.05), suggesting a potential involvement of hsa_circ_101303 in the progression and metastasis of CRC. No significant relationships were identified between hsa_circ_101303 and Ki-67, P53 mutation status, or carcinoembryonic antigen (CEA) levels in CRC (*P* > 0.05), indicating that hsa_circ_101303 might not be directly linked to these specific molecular markers or clinicopathological attributes in CRC.

### Diagnostic value of hsa_circ_101303 in blood on CRC

**Fig. 4:**
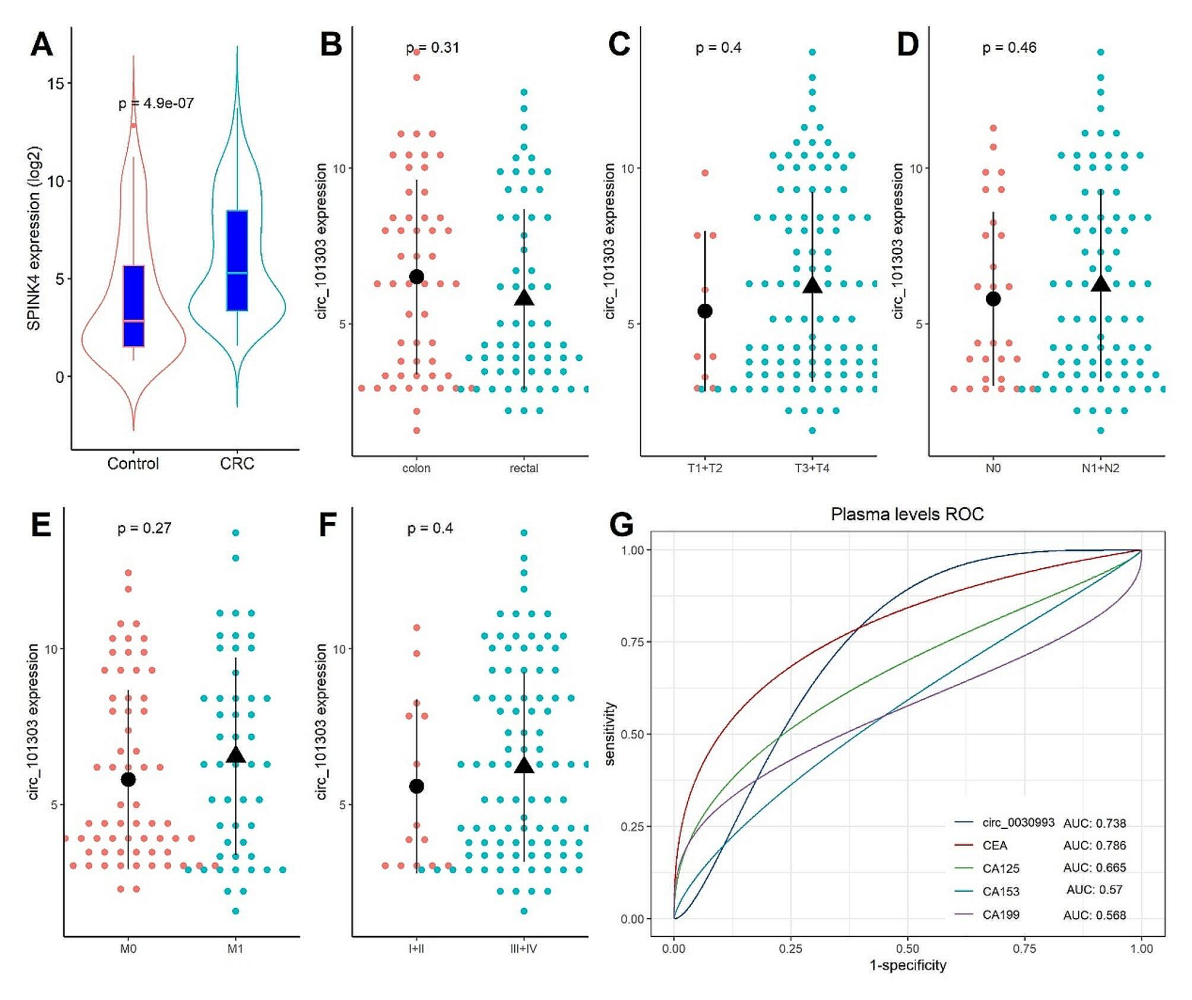
Plasma levels of hsa_circ_101303 in CRC. **(A)** Comparison of plasma levels of hsa_circ_101303 between CRC patients and Controls; **(B-F)** Association of plasma levels of hsa_circ_101303 between CRC patients and clinical parameters; **(G)** Diagnostic value of plasma of hsa_circ_101303 on CRC. Each dot represents a sample value. Data was shown as mean ± SD.

The diagnostic value of hsa_circ_101303 in CRC was assessed by measuring its levels in plasma samples from CRC patients and non-cancer controls through RT-qPCR assay. As depicted in Fig. 4A, plasma levels of hsa_circ_101303 were significantly higher in CRC patients compared to those in non-cancer patients (*P* < 0.05). Examination of the relationship between plasma hsa_circ_101303 levels and clinical parameters indicated no significant differences concerning tumor location, TNM stage, and tumor stage (Fig. 4B-F).

The ROC analysis was employed to evaluate the diagnostic utility of plasma hsa_circ_101303 levels in CRC. In comparison with established tumor biomarkers including CEA, CA125, CA199, and CA153. The AUC values were 0.786 for CEA, 0.665 for CA125, 0.570 for CA153, and 0.568 for CA199, indicating generally lower diagnostic efficacy for CRC. In contrast, hsa_circ_101303 exhibited a moderate diagnostic potential with an AUC value of 0.738, surpassing CA125, CA153, and CA199, yet remaining slightly below CEA (Fig. 4G). These results suggest the feasibility of plasma hsa_circ_101303 as an adjunct diagnostic biomarker for CRC.

### Analysis of the host gene (*LAMP1*) of hsa_circRNA101303 in CRC

The host gene of hsa_circ_101303 was identified as *LAMP1*. Then, we investigated the association between *LAMP1* expression and the clinical parameters of CRC using the TCGA-COADREAD dataset. Using the median value of *LAMP1* expression as a cut-off, we found that high expression of *LAMP1* was associated with N and M stage and tumor stage, as also demonstrated for hsa_circRNA101303. In addition, high expression of *LAMP1* was also associated with patient age and T stage (Table 2).

**Table 2:** Association of LAMP with CRC clinical parameters

	Number (*N* = 635)	High-expression (*N* = 317)	low-expression (*N* = 318)	*P* value
Age	66.4 (12.7)	65.1 (12.8)	67.8 (12.6)	0.008
Gender				0.292
Female	296 (46.6%)	138 (43.5%)	158 (49.7%)	
Male	336 (52.9%)	177 (55.8%)	159 (50.0%)	
Location				0.309
COAD	469 (73.9%)	228 (71.9%)	241 (75.8%)	
READ	166 (26.1%)	89 (28.1%)	77 (24.2%)	
Histological type				0.039
Adenocarcinoma	542 (85.4%)	280 (88.3%)	262 (82.4%)	
Mucinous	79 (12.4%)	29 (9.15%)	50 (15.7%)	
T stage				0.015
T1	21 (3.31%)	6 (1.89%)	15 (4.72%)	
T2	108 (17.0%)	43 (13.6%)	65 (20.4%)	
T3	430 (67.7%)	223 (70.3%)	207 (65.1%)	
T4	72 (11.3%)	42 (13.2%)	30 (9.43%)	
N stage				<0.001
N0	360 (56.7%)	149 (47.0%)	211 (66.4%)	
N1	152 (23.9%)	94 (29.7%)	58 (18.2%)	
N2	117 (18.4%)	69 (21.8%)	48 (15.1%)	
NX	2 (0.31%)	2 (0.63%)	0 (0.00%)	
M stage				<0.001
M0	469 (73.9%)	208 (65.6%)	261 (82.1%)	
M1	88 (13.9%)	52 (16.4%)	36 (11.3%)	
MX	66 (10.4%)	52 (16.4%)	14 (4.40%)	
Tumor stage				<0.001
I	108 (17.0%)	42 (13.2%)	66 (20.8%)	
II	233 (36.7%)	95 (30.0%)	138 (43.4%)	
III	182 (28.7%)	109 (34.4%)	73 (23.0%)	
IV	89 (14.0%)	52 (16.4%)	37 (11.6%)	

### Construction of a ceRNA network for hsa_circRNA101303 in CRC

**Fig. 5:**
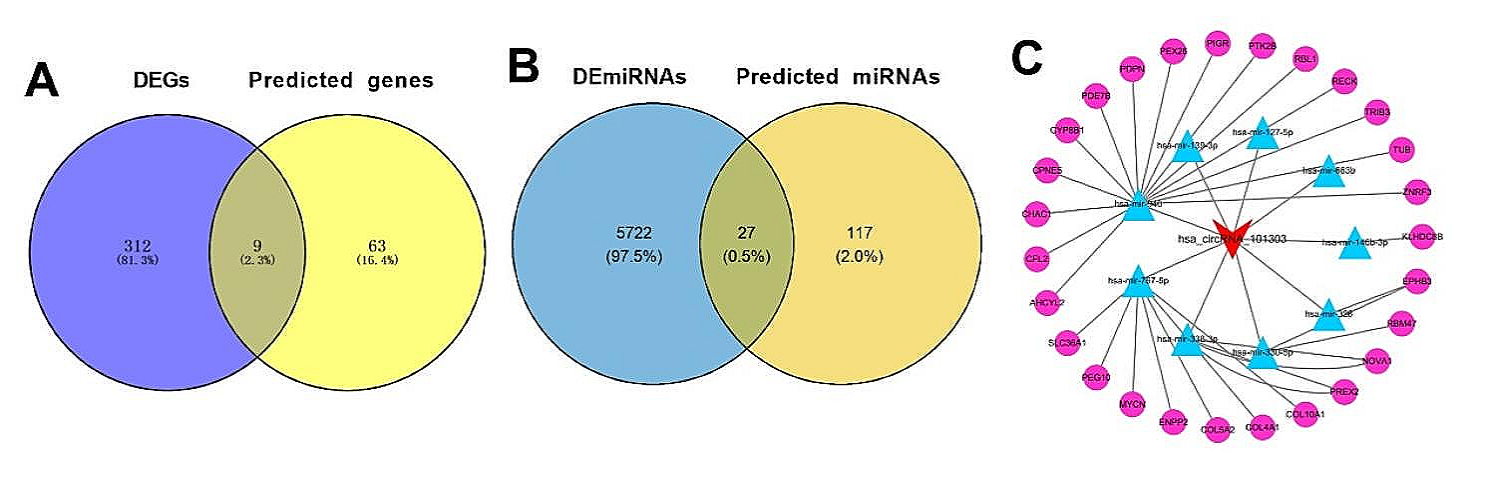
Construction of ceRNA for hsa_circRNA101303 in CRC. **(A)** Intersect miRNAs between DEmiRNAs and predicted miRNAs; **(B)** Intersect genes between DEGs and predicted genes; **(C)** ceRNA network for hsa_circRNA101303 in CRC. Read arrow indicated hsa_circRNA101303, blue triangle indicated miRNAs; purple dot indicated genes. ceRNA: competitive endogenous RNA; DEGs: differentially expressed genes

To elucidate the role of hsa_circ_101303 in CRC, a ceRNA network was constructed utilizing the TCGA-COADREAD dataset. Initially, 282 DEmiRNAs were pinpointed between CRC tumor and normal tissues with a significance threshold of *P* < 0.05. Subsequently, we employed the CircInteractome online tool to predict target miRNAs of hsa_circ_101303, leading to the identification of 72 potential target miRNAs associated with hsa_circ_101303. The overlap between these target miRNAs and DEmiRNAs unveiled nine miRNAs that were both dysregulated in CRC and targets of hsa_circ_101303, forming distinct circRNA-miRNA pairs (Fig. 5A).

Following this, target genes of the identified nine miRNAs were predicted using the miRWalk online tool, resulting in the recognition of 73 target genes. Subsequently, we screened for DEGs between CRC tumor and normal tissues within the TCGA-COADREAD dataset based on defined criteria, culminating in the identification of 1867 DEGs. The intersection of the target genes of miRNAs with the DEGs revealed 27 miRNA-gene pairs (Fig. 5B).

Lastly, by merging the nine circRNA-miRNA pairs with the 27 miRNA-gene pairs, we assembled a comprehensive ceRNA network delineating the regulatory landscape of hsa_circ_101303 in CRC. Visualization of the intricate miRNA-gene interactions within this ceRNA network was achieved using Cytoscape software, as shown in Fig. 5C. This ceRNA network offers valuable insights into the potential regulatory functions of hsa_circ_101303 in the context of CRC.

## Discussion

In this study, we conducted a comprehensive analysis of hsa_circ_101303 in CRC. We initially identified hsa_circ_101303 as significantly upregulated in CRC tissues compared to normal tissues, which was confirmed in multiple datasets. Additionally, the expression of hsa_circ_101303 was elevated in HCC and CCRC, suggesting its potential role as an oncogene across different cancers. We validated the increased expression of hsa_circ_101303 in CRC using independent datasets and clinical samples. Moreover, we explored the clinical relevance of hsa_circ_101303 and observed significant associations with N stage, M stage, and tumor stage, suggesting its involvement in tumor progression. Next, we found that plasma levels of hsa_circ_101303 were in line with that in tissues, and showed moderate diagnostic value. Furthermore, we investigated the target genes of hsa_circ_101303 through constructing a ceRNA network. This network revealed nine miRNAs and 27 genes that potentially interact with hsa_circ_101303. Among these genes, *LAMP1* was associated with the TNM stage of CRC, indicating its potential role in the disease progression. Overall, our study provides evidence for the upregulation of hsa_circ_101303 in CRC and its association with important clinicopathological parameters. The constructed ceRNA network contributes to understanding the potential biological functions of hsa_circ_101303 in CRC and highlights its potential as a therapeutic target or biomarker in the future.

According to current evidence, circRNAs are a novel class of non-coding RNAs that possess a covalently closed loop structure generated from exon and/or intron circularization, thus leading to the deletion of 5′–3′ polarity and the polyadenylated tail [19, 20]. Although many circRNAs have been shown to be related to various diseases, particularly cancers, there are still many circRNAs that need to be investigated [11]. hsa_circ_101303 is located at 13q34 and the length of its spliced sequence is 2540 bp. No previous study has reported the role of hsa_circ_101303 in disease. Our present results indicated that the levels of hsa_circ_101303 were increased in CRC, HCC and CCRC, and were also associated with the progression of CRC. In addition, the expression of *LAMP1* further confirmed the association of hsa_circ_101303 with the pathogenesis and progression of CRC. Collectively, these results indicate the oncogenic role of this circRNA in CRC and the fact that this circRNA may represent a prognostic indicator of CRC.

CircRNAs display heightened cell and tissue specificity, attributed to their stable closed structure compared to long non-coding RNAs (lncRNAs) and microRNAs (miRNAs). This inherent stability underscores their potential utility as diagnostic biomarkers for cancer [21]. Previous studies have highlighted the diagnostic value of circulating circRNAs across various cancer types, including colorectal cancer (CRC). For instance, Ye et al. [22] revealed that plasma levels of hsa_circ_0000370 exhibit a high diagnostic accuracy for CRC, boasting an AUC of 0.8152. Radanova et al. [23] reported moderated diagnostic values for plasma levels of hsa_circ_0001445, hsa_circ_0003028, hsa_circ_0007915, and hsa_circ_0008717 in CRC, with AUC values of 0.739, 0.693, 0.776, and 0.626, respectively. Furthermore, another study demonstrated that plasma levels of hsa_circ_0006282 hold significant diagnostic potential for CRC, with an AUC of 0.831 [24]. In our investigation, we observed an upregulation of hsa_circ_101303 in both plasma and tissue samples of CRC, indicating its consistent expression in these compartments. Moreover, our findings revealed that plasma levels of hsa_circ_101303 offer moderate diagnostic accuracy for CRC (AUC: 0.738) as determined through RT-qPCR assay. While this aligns with the observations by Radanova et al. [23], our results suggest a slightly lower diagnostic accuracy compared to the studies conducted by Ye et al. [22] and Zhu et al. reports [24]. These collective results highlight the potential of hsa_circ_101303 as a promising diagnostic biomarker for CRC. Nevertheless, further validation of its diagnostic accuracy using larger sample sizes is imperative for conclusive assessment.

Research has underscored the pivotal roles that circRNAs play in disease development through various mechanisms, including acting as miRNA sponges or decoys, protein sponges or decoys, enhancers of protein function, and protein scaffolds [25]. Numerous studies have delved into circRNAs functioning as miRNA sponges to modulate target gene expression [26, 27]. The establishment of ceRNA networks relies on extensive transcriptomic datasets and sophisticated computational analyses. These ceRNA networks, governing circRNA-miRNA-mRNA interactions, have been shown to significantly impact the regulation of diverse diseases, including cancers [28, 29]. The robustness and sensitivity of such networks hinge on factors like data acquisition technologies, biological variability, and the integration of multiple data types [30, 31]. In the present study, we found that nine miRNAs were targets of hsa_circ_101303; of these target miRNAs, eight out of nine miRNAs were found to be associated with the pathogenesis of CRC: hsa-miR-139-3p [32], hsa-miR-146b-3p [25], hsa-miR-338-3p [33], hsa-miR-127-5p [34], hsa-miR-326 [35], hsa-miR-330-5p [36], hsa-miR-663b [37] and hsa-miR-940 [38], thus verifying the robustness of circRNA-miRNAs interactions. Furthermore, hsa-miR-767-5p was also reported to regulate the pathogenesis of gastric cancer [39]. Therefore, our results provide evidence to promote the further investigation of hsa_circ_101303 in CRC.

Our study provides valuable insights into the association of hsa_circ_101303 with CRC; however, there are several limitations that should be acknowledged. Firstly, our analysis focused on the expression of hsa_circ_101303 at the tissue level, but it is important to investigate its expression in peripheral circulation and exosomes as well. Comparing its expression with traditional tumor biomarkers could provide further understanding of its diagnostic and prognostic value. Secondly, the sample size in our study was relatively small, which may limit the generalizability of our findings. Conducting studies with larger cohorts would help validate the results and increase the statistical power. Thirdly, our study primarily relied on bioinformatics analysis and lacked experimental validation at the cellular and animal levels. Fourth, this study utilized statistical methods including Student’s t-test, ANOVA test, and ROC analysis. While these methods were chosen based on the assumptions required by the distribution of variables, each statistical method has its own limitations. Any deviations from these assumptions could affect our results, and thus, our findings should be interpreted with caution. Further research involving functional studies, such as knockdown experiments to elucidate the biological function of hsa_circ_101303 in CRC, would provide deeper insights into its role in tumorigenesis and progression. Considering these limitations, future studies should address these aspects to further evaluate the clinical significance and functional mechanisms of hsa_circ_101303 in CRC.

## Conclusions

Our study provided compelling evidence for the upregulation of hsa_circ_101303 in CRC and its association with disease progression. Moreover, we demonstrated that plasma levels of hsa_circ_101303 have potential as a diagnostic biomarker for CRC. The construction of a ceRNA network for hsa_circ_101303 further enhanced our understanding of its regulatory mechanisms in CRC.

## Data Availability

The data used to support the findings of this study are available from the corresponding author upon reasonable request.
